# Supplementary material for: Blocking IbmiR319a Impacts Plant Architecture and Reduces Drought Tolerance in Sweet Potato
Source: Genes (Basel). 2022 Feb 24;13(3):404. doi: 10.3390/genes13030404 (PMC8953241; doi:10.3390/genes13030404)
Supplement: Supplementary file 1 [file genes-13-00404-s001.zip › genes-1578625-supplementary.pdf]

Supplement

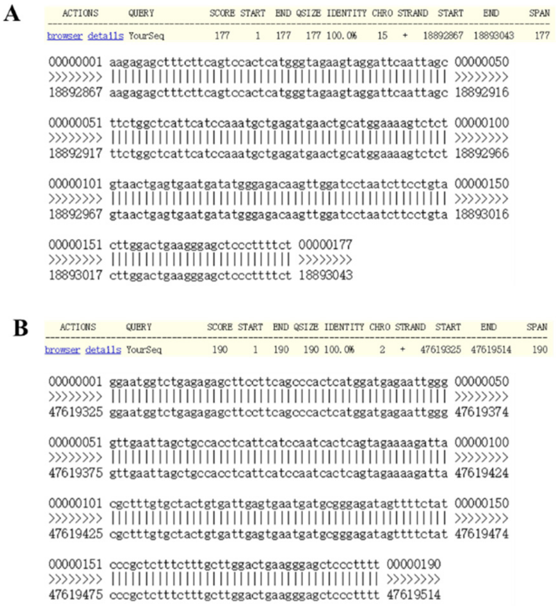

Supplement Figure S1. The results of blasting the *IbmiR319a* precursor (**A**) and *IbmiR319c* precursor (**B**) in the sweet potato public database.

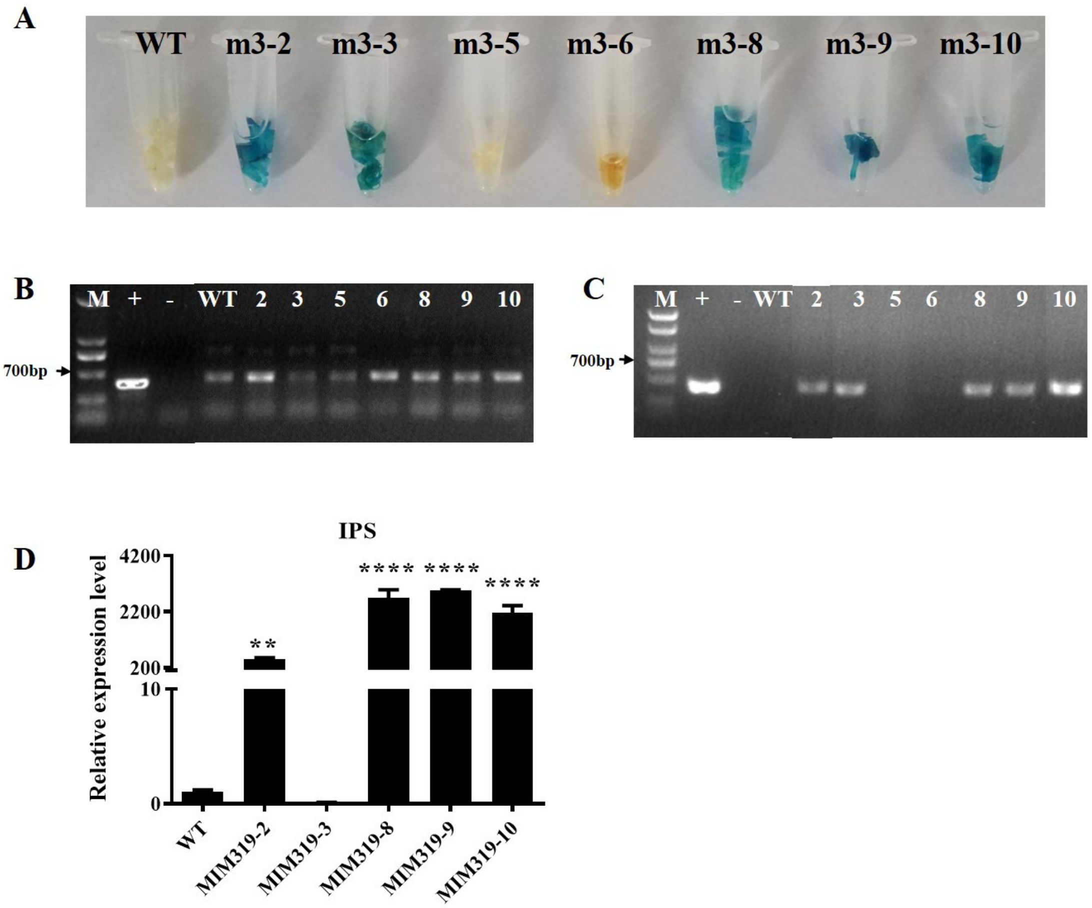

Supplement Figure S2. The detection of MIM319 transgenic sweet potato. **A**: GUS histochemical staining of MIM319 transgenic sweet potato. **B**, **C**: Electrophoresis of PCR products of the *GUS* gene and *IPS* gene. M: molecular weight marker 2000 bp DNA ladder; +: MIM319 plasmid as the positive control; **-**: water as the negative control; 2,3,5,6,8,9,10: transgenic plants. **D**: Expression profiles of the *IPS* gene in MIM319 transgenic plants by qRT-PCR. Data are presented as means ± SE, and error bars represent the SE. Asterisks indicate significant differences between transgenic and control plants at $P<0.01$ by Student's *t*-test.

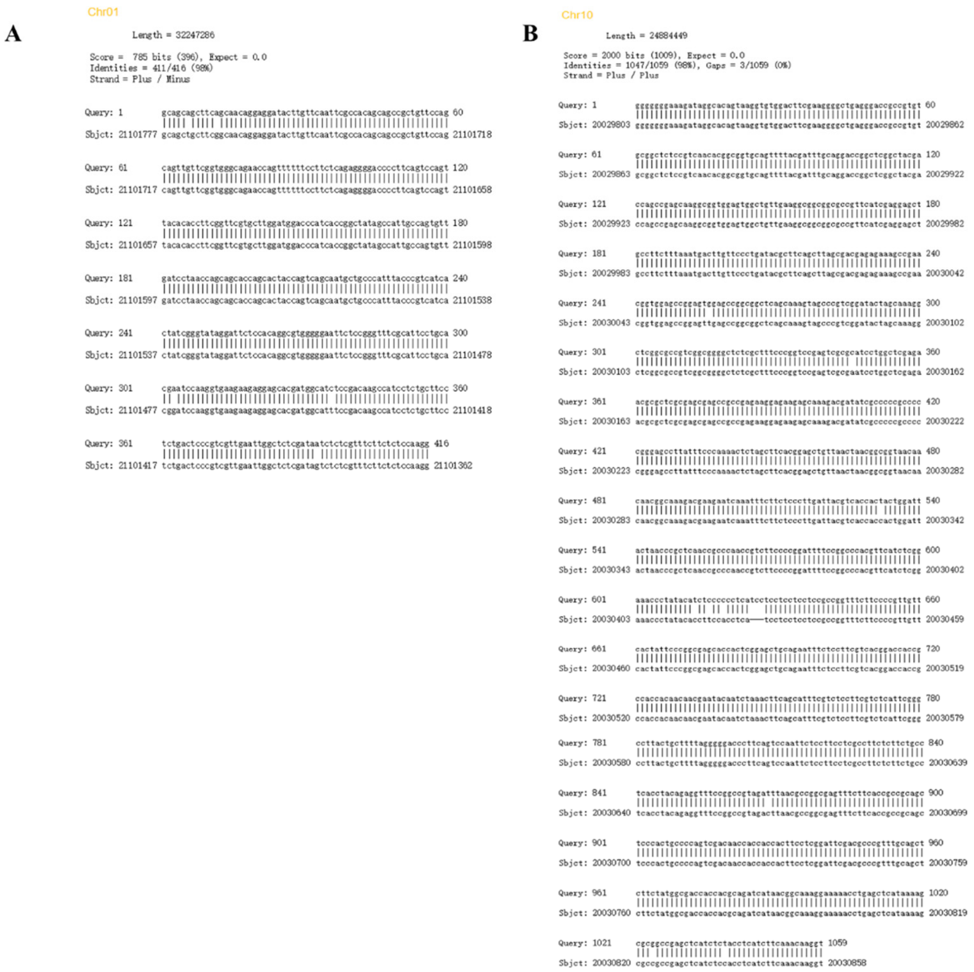

Supplement Figure S3. The results of the blast search of the target genes *IbTCP11* (**A**) and *IbTCP17* (**B**) in the genomics database of the two wild ancestors (*I. trifida and I. triloba*) of sweet potato.

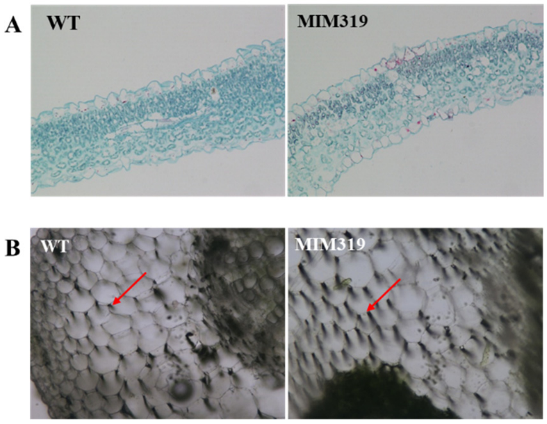

Supplement Figure S4. Microscopic analysis of the leaf transverse paraffin section (**A**) and petiole transverse freehand section (**B**) in MIM319 transgenic sweet potato and WT. The red arrow shows the intercellular space.

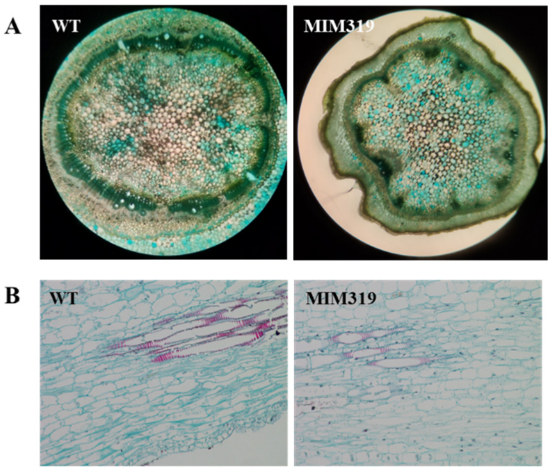

Supplement Figure S5. Lignin deposition patterns in the stem of the third internode freehand transverse section staining with toluidine blue (TB) (**A**) and vertical paraffin section staining with Safranin O-Fast Green (**B**).

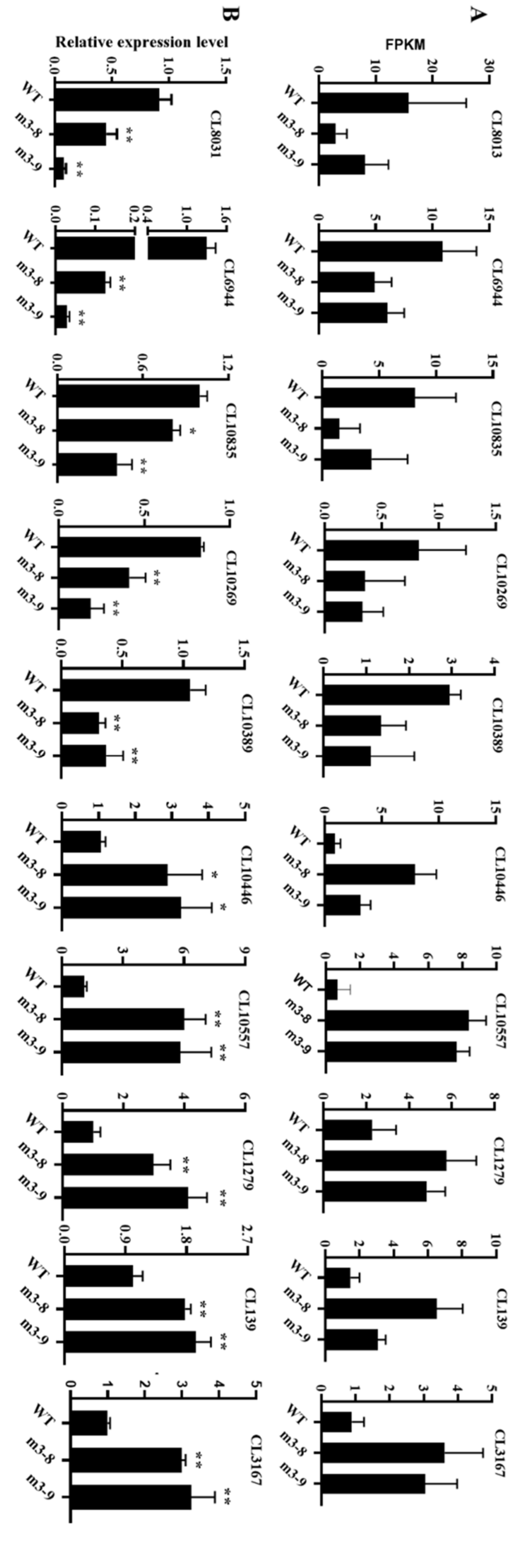

Supplement Figure S6. Validation of the transcriptome sequencing results. Expression patterns of randomly selected differentially expressed genes (DEGs) obtained by sequencing (**A**) and qRT-PCR (**B**). All qRT-PCRs were repeated three times for each sample, and the results are expressed as the mean ± SD. **P* <0 .05 or ***P* <0 .01 were considered statistically significant.
